# Optimization of Precursor Preparation in PSMA-11 Radiolabeling to Obtain a Highly Reproducible Radiochemical Yield

**DOI:** 10.3390/ph15030343

**Published:** 2022-03-11

**Authors:** Antonella Iudicello, Stefano Boschi, Pietro Ghedini, Frank Lohr, Stefano Panareo

**Affiliations:** 1 Pharmaceutical Department, Azienda USL di Modena, Largo del Pozzo 71, 41121 Modena, Italy; 2 Nuclear Medicine Unit, Department of Oncology and Hematology, Azienda Ospedaliero-Universitaria di Modena, Largo del Pozzo 71, 41124 Modena, Italy; ghedini.pietro@aou.mo.it (P.G.); panareo.stefano@aou.mo.it (S.P.); 3 Department for Life Quality Studies, University of Bologna, Corso D’Augusto, 237, 47921 Rimini, Italy; stefano.boschi@unibo.it; 4 Radiotherapy Unit, Department of Oncology and Hematology, Azienda Ospedaliero-Universitaria di Modena, Largo del Pozzo 71, 41124 Modena, Italy; lohr.frank@aou.mo.it

**Keywords:** gallium-68, PSMA-11, radiochemical yield, molar activity, net precursor content, clinical routine

## Abstract

[^68^Ga]Ga-PSMA-11 PET/CT plays a pivotal role in the diagnosis and staging of prostate cancer because of its higher sensitivity and detection rate compared with traditional choline PET/CT. A highly reproducible radiochemical yield of the radiopharmaceutical to be used in the clinical routine is an important parameter for planning and optimization of clinical activity. During radiometallation of PSMA-11, the presence of metal ion contaminants in the peptide precursor may cause a decrease in the [^68^Ga]Ga-PSMA-11 radiochemical yield because of metal ion contaminants competition with gallium-68. To optimize the radiochemical yield of [^68^Ga]Ga-PSMA-11 radiosynthesis, data obtained by preparing the solution of the PSMA-11 precursor with three different methods (A, B, and C) were compared. Methods A and B consisted of the reconstitution of different quantities of precursor (1000 µg and 30 µg, respectively) to obtain a 1 µg/mL solution. In Method A, the precursor solution was aliquoted and stored frozen, while the precursor solution obtained with Method B was entirely used. Method C consisted of the reconstitution of 1000 µg of precursor taking into account net peptide content as described in European Pharmacopoeia. Radiosynthesis data demonstrated that reconstitution methods B and C gave a consistently higher and reproducible radiochemical yield, highlighting the role of metals and precursor storage conditions on the synthesis performance.

## 1. Introduction

Chemical precursors for radiopharmaceutical preparations are non-radioactive substances obtained by chemical synthesis to be combined with a radionuclide (in contrast to precursors manufactured using substances of human or animal origin) [1].

Chemical precursors require an adequate characterization, according to the general requirements, as a part of their quality assurance in order to demonstrate the safety and efficacy of the final radiopharmaceutical preparation [1,2].

Stability testing is part of the chemical precursor’s characterization. The purpose of stability testing is to provide evidence on how the quality of a substance varies with time under the influence of a variety of environmental factors such as temperature, humidity, and light, and to establish a re-test period and recommended storage conditions [3].

**Figure 1:**
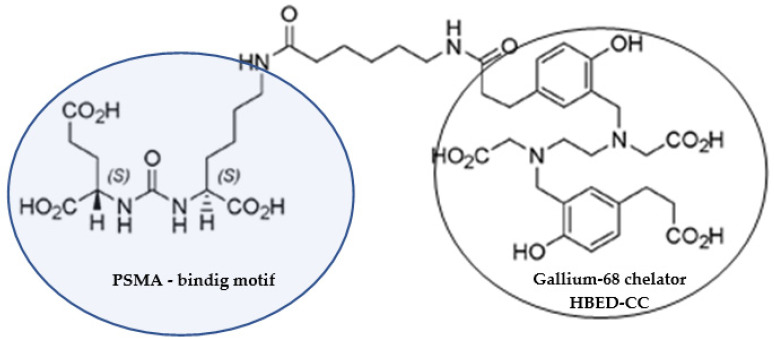
Chemical structure of PSMA-11.

The PSMA (prostate-specific membrane antigen) conjugated to HBED-CC (N,N’-bis [2-hydroxy-5-(carboxyethyl)benzyl]ethylenediamine-N,N’-diacetic acid), better known as PSMA-11, is one of the most widespread precursors for PET imaging of prostate cancer after radiolabeling with gallium-68 because the PSMA is highly expressed on most prostate cancer (PCa) cells (Figure 1).

Stability testing for the PSMA-11 has provided evidence that the quality of precursor in aqueous solution varies with time due to the presence of Fe (III) in the precursor material and the consequent interaction between HBED-CC and Fe (III) already at room temperature [4].

The formation of the complex between HBED-CC and Fe (III) in the PSMA-11 aqueous solutions causes a decrease of the [^68^Ga]Ga-PSMA-11 radiochemical yield (RCY) because the Fe (III) competes with gallium-68 during the complexation reaction with the chelator [4].

Generally, a highly reproducible % RCY of the tracers to be used in the clinical routine is an important parameter for the planning and optimization of clinical activity. Radiochemical M^3+^-L complex formation yields, in most cases, can be increased by providing a higher concentration of the ligand [5].

However, a high molar activity (A_m_) or specific activity (A_s_) of the radiopharmaceutical is preferred, and it is known that if the precursor (ligand) increases, the molar activity decreases [6].

Recent EANM guidelines on harmonization of the molar activity or specific activity of radiopharmaceuticals reported that in the case of radiometallation of peptides the presence in the peptide precursor of metal ion contaminants indirectly affects A_m_ by necessitating higher amounts of peptide precursor to achieving high radionuclide incorporation. For such radiotracers, we are therefore normally referring to the apparent A_m_, empirically determined by dividing the amount of radioactivity present in an exact volume of the final formulation by the amount of peptide precursor used in the labeling process in mol (or µmol) [6,7].

Nevertheless, if a monograph has been published such as e.g., for [^68^Ga]Ga-PSMA-11, the maximum amount of precursor (ligand) to use in the synthesis has already been defined.

In this manuscript, we present the data obtained by preparing the solution of PSMA-11 synthesis precursor following various methods in order to obtain a high % RCY that is also reproducible over time. All tested methods took into account that: generally, a high A_m_ is preferred; the European Pharmacopoeia (Ph. Eur.) Monograph Gallium (^68^Ga) PSMA-11 injection (3044) prescribes to use of a maximum of 30 µg of PSMA-11 for [^68^Ga]Ga-PSMA-11 synthesis [8]; the Ph. Eur. monographs are referred to net precursor content, and the lyophilized PSMA-11 provided by the manufacturer contains also water, counter ions, and residual solvents [3].

The net peptide content is the percentage of peptide material in the lyophilisate.

## 2. Materials and Methods

### 2.1. Chemicals and Reagents

Gallium-68 (t_1/2_ = 68 min, β + = 89%, and EC = 11%) for the [^68^Ga]Ga-PSMA-11 production, was routinely obtained as gallium-68 chloride ([^68^Ga]GaCl_3_) solution from a pharmaceutical-grade 1.85 GBq germanium-68/gallium-68 generator (GalliaPharm^®^, Eckert&Ziegler Radiopharma GmbH, Berlin, Germany).

Sterile and ultrapure 0.1 M Hydrochloric Acid (HCl) for the elution of germanium 68/gallium-68 generator was purchased from Eckert & Ziegler Radiopharma GmbH (Berlin, Germany).

Synthesis was performed on a fully automated radiosynthesis cassette module (GAIA V2™, Elysia-Raytest, Straubenhard, Germany), operating in a laminar flow isolator class A.

The detectors of the synthesis module were calibrated with a dose calibrator (ISOMED 2010, MED Nuklear-Medizintechnik Dresden GmbH, Dresden, Germany) as reference.

PSMA-11 lyophilized vials for [^68^Ga]Ga-PSMA-11 radiosynthesis, ^nat^Ga-PSMA-11 reference standard for [^68^Ga]Ga-PSMA-11, disposable sterile cassettes (SCX fluidic kit for the [^68^Ga]Ga-labeling of peptides), and disposable reagent kits (SCX reagent kit for the [^68^Ga]Ga-labeling of peptides) containing all consumables necessary for gallium-68 radiolabelling of peptides except peptide, all were of GMP grade and were purchased from Advanced Biochemical Compounds, ABX (Radeberg, Germany).

Ammonium Acetate, Methanol, and Acetonitrile (ACN) were purchased from Carlo Erba Reagents S.r.l. (Cornaredo, Milan, Italy); Trifluoroacetic Acid (TFA) and metal-free water (Fluka Water Trace-Select^®^ for trace Analysis) were purchased from Merck Life Science S.r.l. (Milan, Italy); Ultrapure water (Milli-Q, 18.2 MΩ) was obtained from a Milli-Q^®^ IQ Element purification (Merck KGaA, Darmstadt, Germany). All chemicals were of analytical grade and they were used without further purification. HPLC eluents (Milli-Q water, ACN, and TFA) were of high-grade purity.

### 2.2. Precursor Preparation and Data Collection

The aqueous solution of PSMA-11 synthesis precursor (1 µg/µL) for [^68^Ga]Ga-PSMA-11 radiosynthesis was obtained as follows:

- reconstituting a 1000 µg vial of PSMA-11 precursor for [^68^Ga]Ga-PSMA-11 with 1000 µL of metal-free water (hereinafter Method A);
- reconstituting a 30 µg vial of PSMA-11 precursor for [^68^Ga]Ga-PSMA-11 with 30 µL of metal-free water (hereinafter Method B);
- reconstituting a 1000 µg vial of PSMA-11 precursor for [^68^Ga]Ga-PSMA-11 with 840 µL of metal-free water (hereinafter Method C), considering that the percentage of peptide material in the lyophilisate (i.e., net precursor content) was 84% according to CoA provided by the manufacturer.

The precursor solutions obtained with Methods A and C were immediately aliquoted (30 µL) in 0.5 mL Eppendorf tubes and frozen at −25 °C. For method B, the precursor solution was reconstituted just before starting the synthesis.

To perform the [^68^Ga]Ga-PSMA-11 synthesis, 30 µL of precursor solution (1 µg/µL) was used. Each method described was used ten times within 1–2 months. 

Since 30 µg vials of PSMA-11 precursor were supplied in a box of 5 vials × 30 µg and one vial was used to prepare the reference solution b in order to perform the quality control of the [^68^Ga]Ga-PSMA-11 injectable solutions according to Ph. Eur. Monograph Gallium (^68^Ga)PSMA-11 injection (3044) [8], Method B was used nine times.

### 2.3. Automated [^68^Ga]Ga-PSMA-11 Synthesis

Synthesis was performed utilizing the standard radiolabelling method [9,10,11,12]. An aliquot of 30 μL of the aqueous solution of PSMA-11 synthesis precursor (1 μg/μL), taken with a precision micropipette (Pipetman^®^ Gilson, variable volume 10–100 μL), was radiolabelled in 3.00 ± 0.2 mL ammonium acetate buffer (0.08 M; reagent kit) and 550.0 ± 78 μL eluent (reagent kit) with 785 ± 323 MBq gallium-68. After radiolabelling (90 ± 2 °C; 250.0 ± 13 s), the reaction mixture was passed over a C18 cartridge (reagent kit) and washed with sterile water for injection (reagent kit). The purified product was eluted with 1.5 mL 60 vol% ethanol (reagent kit) and 15 mL saline solution (0.9% sodium chloride) followed by sterile filtration to obtain the final formulation.

### 2.4. Quality Control

For quality control, an aliquot of 100 μL was retained from the final product before measurement of the radioactivity.

The quality control was performed according to the specifications given by the Ph. Eur. in the Monograph Gallium (^68^Ga) PSMA-11 injection (3044) [8].

Radiochemical purity (RCP), Chemical purity (CP), and identification of the product species were determined using radioHPLC analysis. Additionally, RCP was also determined by radio thin-layer chromatography (radioTLC).

Thin-layer chromatography was performed using a glass microfiber chromatography paper impregnated with silica-gel (iTLC-SG, Agilent Technologies Italia Spa, Cernusco Sul Naviglio, Italy) developed in 1 M ammonium acetate/methanol (1:1) and analyzed using a single trace radio TLC-scanner (PET-miniGita, Elysia-Raytest, Straubenhardt, Germany) and evaluation software (Gina Star TLC, Elysia-Raytest, Straubenhardt, Germany).

RadioHPLC analysis was performed on a Thermo Scientific Dionex Ultimate 3000 HPLC system (Thermo Scientific, Bremen, Germany) equipped with LPG-3400SD pump, TCC-3000 column oven, UV VWD-3100 detector, and radiometric detector at NaI (Gabi Star, Elysia-Raytest, Straubenhardt, Germany) connected in series. Reversed-Phase High-Performance Liquid Chromatography (RP-HPLC; ACE 3 μm C18, l = 0.6 m, Ø = 7 mm; Thermo Scientific, Bremen, Germany) with a linear A–B gradient (0–0.5 min 5% B, 0.5–10 min 5% B to 40% B, 10–11 min 40% B to 5% B, 11–16 min 5% B) at a flow rate of 0.6 mL/min and a total run time of 16 min was performed. Solvent A consisted of 0.1% TFA in Milli-Q water and solvent B of 0.1% TFA in ACN.

UV absorbance was measured at 280 nm. The column temperature was kept at 24 °C. The injection volume was 20 μL. The Chromeleon data system software (Version 7.2.8) was used for data acquisition and mathematical calculations. 

The approximate half-life of gallium-68 was determined using the dose calibrator (ISOMED 2010, MED Nuklear-Medizintechnik Dresden GmbH, Dresden, Germany).

For radionuclidic identification and determination of germanium-68 breakthrough, the energy of gamma photons was measured using a CZT-based gamma-ray detector designed and produced by KromekTM (Sedgefield, County Durham, UK) [13] and evaluation software (MultiSpect Analysis gamma spectroscopy software version 22.2, Sedgefield, County Durham, UK). 

The appearance was checked visually. pH was measured using micro-electrode and a millivoltmeter (pH meter) from Thermo Scientific™ Orion™ Dual Star. 

Bacterial endotoxins were evaluated with Endosafe^®^ Portable Testing System™ (PTS™) portable system for endotoxin testing (Charles River, Charleston, SC, USA). The method used to check the filter integrity was the bubble point test, which was automatically carried out by the radiosynthesis module to reduce the radiation exposure of the operators.

### 2.5. Radiochemical Yield (RCY) and Radioactivity at EOS Determination

Performance evaluation of the used methods was performed in terms of % RCY and activity at the end of the synthesis (EOS). 

RCY was calculated based on the radioactivity of the final product vial, measured using a dose calibrator (ISOMED 2010, MED Nuklear-Medizintechnik Dresden GmbH, Dresden, Germany), and the radioactivity of the eluate of gallium-68 collected at the end of a generator elution performed 24 h before the radiosynthesis and measured with the dose calibrator. This value was decay-corrected at EOS.

The radioactivity at EOS was the amount of radioactivity of the final product calculated using the dose calibrator and expressed in MBq.

All data obtained from routine clinical production were retrospectively analyzed.

### 2.6. Statistical Analysis

All data were expressed as mean ± standard deviation (S.D.) and percentage of coefficient of variation (% CV) to monitor the reliability and reproducibility of methods, and they were reported in a summary table (Table 1). The coefficient of variation should not exceed 5% (acceptance criterion). 

To compare the different methods a two-sample *t*-test with a significance level of α = 0.05 (95% of confidence interval) was used. The null hypothesis assumes that no statistically significant difference exists between the methods means.

## 3. Results

Table 1 shows [^68^Ga]Ga-PSMA-11 synthesis data obtained from three different methods of precursor preparation.

The data set includes data from all batches of [^68^Ga]Ga-PSMA-11 produced consecutively and starting from the day of precursor solution preparation for Methods A and C. None of the syntheses was excluded. 

**Figure 2:**
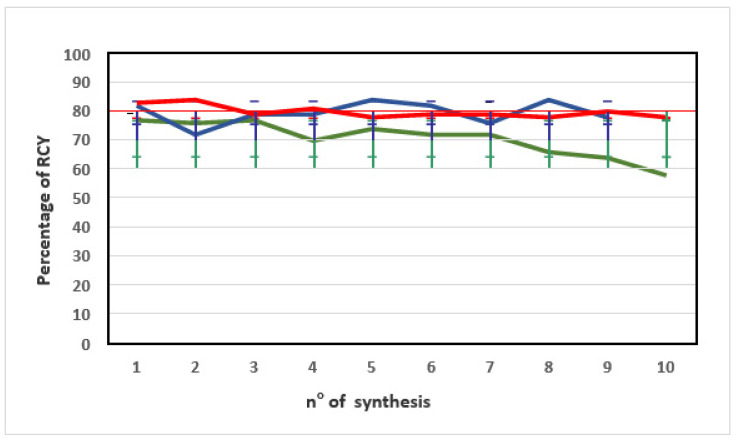
Trend of the percentage of RCY obtained from [^68^Ga]Ga-PSMA-11 radiosyntheses performed using a PSMA-11 precursor solution from methods A (green line), B (blue line), and C (red line). The error bars show standard deviations (SD).

The three methods led to different results in terms of repeatability of % RCY. The starting % RCY was similar for all methods. With time, the % RCY decreased for [^68^Ga]Ga-PSMA-11 syntheses performed preparing the precursor solution with Method A, while it was almost constant for Methods B and C (Figure 2).

Since the average starting activities (MBq) of eluted gallium-68 were not in the same range for the different methods (Table 1), we analyzed and compared % RCY data obtained with the same method (Method C) utilizing a germanium-68/gallium-68 generator (1.85 GBq) at different shelf lives (Table 2).

The analysis of variance (ANOVA) allowed evaluating the % RCY data with the post-Fischer test using a significance level of α = 0.05 (95% of confidence interval). As shown in Table 2, the calculated F-value (1.5502) is lower than the tabulated F-value (3.35), indicating no statistically significant difference between % RCY data obtained from Method C utilizing a germanium-68/gallium-68 generator at the end, at the start, or in the middle of shelf life (*p* > 0.05). It can be assumed that this result may be transferred to other methods.

### 3.1. Quality Control

Quality control was performed according to the specifications provided by the Ph. Eur. Monograph Gallium (^68^Ga) PSMA-11 injection (3044) [8]. For all preparations, the specifications were always met. Particularly, the % RCP of the final products was determined with >95% on average independent from the precursor preparation method (Table 3). 

In UV chromatograms from the test solutions of [^68^Ga]Ga-PSMA-11, the sum of the areas of the peaks due to compounds with a relative retention of 0.8 to 1.3 with reference to a 3 μg/mL reference solution of PSMA-11 (reference solution b) were always not more than the area of the principal peak in UV chromatogram of reference solution b (radiochemical purity) [8]. 

**Figure 3:**
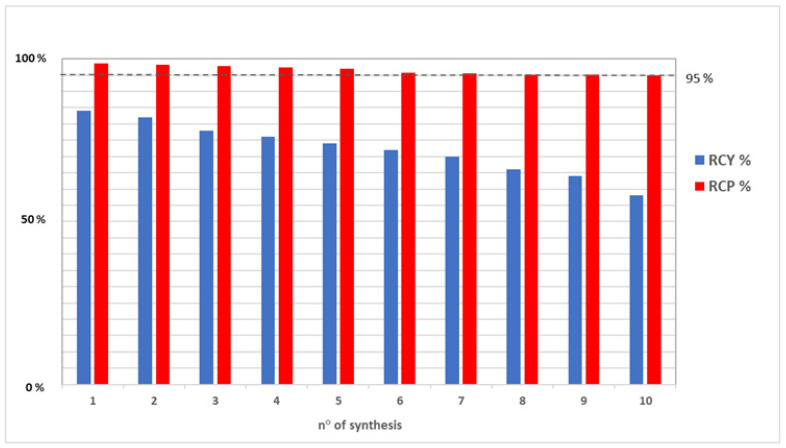
Comparison between % RCY (blue bars) and % RCP (red bars) data from [^68^Ga]Ga-PSMA-11 preparations: a decrease of % RCY corresponds to a decrease in % RCP close to the lower limit of the pharmacopeia specifications. The dotted line represents this minimum acceptable value of % RPC (95%).

Table 3 shows that the % RCY decrease corresponds to an increase in the unspecified impurities into [^68^Ga]Ga-PSMA-11 preparations (see residual in C18 cartridge), as well as to an increase in gallium-68 in colloidal and ionic forms and, consequently, to a decrease in % RCP close to the lower limit of the pharmacopeia specifications (Method A). Indeed, for radiotracers clinical use, it is mandatory to obtain radiochemical purities ≥ 95% [6]. This is shown in Figure 3, where the decrease of blue bars (% RCY) corresponds to a decrease of red bars (% RCP).

### 3.2. Statistical Analysis

Comparing the % CV between % RCY data, a % CV less than 5% (acceptance criterion) was found for Method B and C, not for Method A (Table 1).

This shows that only the % RCY data from Method B and C are repeatable according to ICH guidelines [14]. 

The difference between % RCY obtained with Methods A and C as well as the difference between % RCY obtained with Methods A and B were statistically significant (calculated *t*-value is greater than the tabulated *t*-value). No statistically significant difference was found between % RCY obtained with Methods B and C (0.24 < 2.11. Table 4).

## 4. Discussion

In the present study, we analyzed and compared the [^68^Ga]Ga-PSMA-11 synthesis data obtained by performing the preparation of PSMA-11 precursor solution with different methods. 

According to Ph. Eur. Monograph Gallium (^68^Ga) PSMA-11 injection, a maximum of 30 µg of PSMA-11 for [^68^Ga]Ga-PSMA-11 synthesis is allowed [8].

PSMA-11 precursor for [^68^Ga]Ga-PSMA-11 is supplied as a 1000 µg/vial or a 30 µg/vial (box of 5 vials), therefore precursor solution of PSMA-11 (1 µg/µL) is commonly obtained reconstituting the 1000 µg vial of PSMA-11 with 1000 µL of metal-free water, then aliquoting (30 µL) and freezing the solution (Method A). Alternatively, it could be obtained by reconstituting the 30-µg vial with 30 µL of metal-free water just before starting [^68^Ga]Ga-PSMA-11 synthesis (Method B).

During the routine clinical production of [^68^Ga]Ga-PSMA-11, we observed a decrease of % RCY, and consequently of the radioactivity at the EOS, if the preparation of precursor solution was performed according to Method A. Within one month from precursor solution preparation, % RCY decreased from 77% to 58% and the radioactivity at the EOS from 570 MBq to 401 MBq (Table 1, left column). Assuming 70-kg patients and administering 2-MBq/kg body weight, over one month one patient dose was lost.

Previously, we described that the stability of PSMA-11 precursor in aqueous solution is poor due to the presence of Fe (III) in the PSMA-11 starting material [4]. The interaction between HBED-CC and Fe (III) in aqueous environment already at room temperature and the consequent formation of side product Fe(III)-PSMA-11 cause, over time, a decrease in the PSMA-11 available to gallium-68 complexation. This is reflected in a decrease in % RCY [4]. Indeed, with time a decrease in the % RCY was not observed if precursor solution was obtained just before starting [^68^Ga]Ga-PSMA-11 synthesis (Table 1, Method B). 

The % CV between % RCY from Method B was <5%, indicating the repeatability of the method.

Considering that the lyophilized PSMA-11 provided by the manufacturer contains water, counter ions, and residual solvents and that generally the Ph. Eur. Monographs refer to net precursor content (i.e., to the percentage of precursor in the lyophilisate), we prepared the PSMA-11 precursor solution (1 µg/µL) reconstituting a 1000 µg vial of PSMA-11 precursor for [^68^Ga]Ga-PSMA-11 with 840 µL of metal-free water (Method C). Indeed, according to CoA provided by the manufacturer, the percentage of PSMA-11 in the lyophilisate (i.e., net precursor content) was 84%. 

Figure 2 shows that preparing the PSMA-11 precursor solution with Method C, with time % RCY remained almost constant, as confirmed by the low % CV (<3%) (Table 1). 

It is important to note that since the data set included data from ten batches of [^68^Ga]Ga-PSMA-11 produced consecutively via each Method A, B, or C, starting from the day of precursor preparation for Methods A and C, a substantial decrease in % RCY was reported within 1 month for Method A, while % RCY remained almost constant within 2 months for Methods B and C (Table 1). 

These results are in agreement with data previously reported by our group [4] according to which the increase of the amount of PSMA-11 precursor available to gallium-68 complexation (Method C) or the use of a freshly prepared PSMA-11 precursor solution (Method B) reduces the presence of unspecified impurities (residual in C18 cartridge) as well as gallium-68 in colloidal and ionic forms in the final product, which, conversely, increases over time using Method A (Table 3). Indeed, the *t*-tests showed no statistically significant difference between % RCY obtained with Methods B and C (Table 4), despite that, using Method B, the net peptide content is less than using Method C. This occurs because the affinity of HBED-CC for both Ga (III) and Fe (III) is similarly high (log KGa = 37.73, log KFe = 36.74) [15,16]. Therefore, according to Iudicello et al. [4], using a precursor solution freshly prepared (Method B), the competition between Fe (III) and gallium-68 during the complexation reaction with HBED-CC is less than using a precursor solution not freshly prepared (Method A), because of the time-dependent formation of the complex between Fe (III) and HBED-CC during storage. Differently, it can be assumed that using a precursor solution more concentrated (Method C), the availability of HBED-CC for the complexation with the gallium-68 is similar to the use of a precursor solution freshly prepared (Method B).

Both methods B and C are superior to Method A in providing the [^68^Ga]Ga-PSMA-11 radiopharmaceutical with high and highly reproducible % RCY, but Method C is more feasible than Method B because it reduces the cost for precursor (1 vial × 1000 µg cost less than 5 vials × 30 µg and it is available for 20 syntheses) and permits to prepare the precursor solution once a month instead of just before starting all syntheses.

Regarding the apparent A_m_, since the difference in the precursor micrograms used in the different methods is very small, it can be excluded that the higher concentration of the peptide used in Method C can cause a significant decrease in the apparent A_m._

## 5. Conclusions

The contamination from starting materials during the radiosynthesis can dilute the radiolabelled compound with its non-labeled analog and cause a decrease of % RCY [6].

This occurs also in the radiomettallation of PSMA-11 where the presence of Fe (III) in the precursor material and the consequent interaction between HBED-CC and Fe (III) in aqueous environment already, at room temperature, causes a decrease of the [^68^Ga]Ga-PSMA-11 RCY [4].

In the present study, [^68^Ga]Ga-PSMA-11 synthesis data obtained preparing the solution of PSMA-11 precursor following various methods in order to obtain a highly reproducible RCY were analyzed.

Our data demonstrate that it is possible to obtain over time a high (81 ± 3% non-decay corrected) and highly reproducible RCY either using a PSMA-11 precursor solution reconstituted just before starting the synthesis or preparing the solution of the precursor up to two months earlier starting the synthesis. In this case, however, the net PSMA-11 content in the lyophilisate for the precursor reconstitution must be considered.

No statistical difference between % RCY obtained with these two methods was observed; however, the second method (Method C) is more feasible in terms of cost, reliability, and preparation time.

## Figures and Tables

**Table 1:** Comparison of [^68^Ga]Ga-PSMA-11 synthesis data from three methods.

	Method A			Method B			Method C		
**Generator calibration date**	2020/08/11 **^a^**			2020/08/11 **^b^**			2020/08/11 **^c^**		
**Precursor reconstitution date**	2020/11/10			Before starting synthesis			2021/03/16		
**Process number**	**Date**	**RCY, % ^d^**	**Radioactivity** **at EOS, MBq ^d^**	**Date**	**RCY, % ^d^**	**Radioactivity** **at EOS, MBq ^d^**	**Date**	**RCY, % ^d^**	**Radioactivity** **at EOS, MBq ^d^**
1	2020/11/10	**77**	570	2021/02/09	**82**	482	2021/03/16	**83**	442
2	2020/11/11	**76**	560	2021/02/10	**72**	422	2021/03/17	**84**	448
3	2020/11/12	**77**	567	2021/02/16	**79**	460	2021/03/24	**79**	416
4	2020/11/17	**70**	512	2021/02/17	**79**	457	2021/03/30	**81**	411
5	2020/11/18	**74**	537	2021/02/23	**84**	478	2021/03/31	**78**	401
6	2020/11/19	**72**	520	2021/03/02	**82**	460	2021/04/07	**79**	399
7	2020/11/24	**72**	511	2021/03/03	**76**	425	2021/04/21	**79**	387
8	2020/12/01	**66**	465	2021/03/09	**84**	456	2021/04/22	**78**	380
9	2020/12/02	**64**	448	2021/03/10	**78**	426	2021/05/05	**80**	372
10	2020/12/10	**58**	401		**n.d.**	**n.d.**	2021/05/12	**78**	360
**Means**		**70.6**	**509.1**		**79.6**	**451.8**		**79.9**	**401.6**
**SD**		**6.24**	**55.70**		**3.94**	**22.50**		**2.13**	**28.62**
**CV%**		**8.84**			**4.95**			**2.67**	

^a^ Germanium-68/gallium-68 generator at the start of shelf life. ^b^ Germanium-68/gallium-68 generator in the middle of shelf life. ^c^ Germanium-68/gallium-68 generator at the end of shelf life. ^d^ Non-decay corrected. **Notes:** As shown in Table 1, assuming 70-kg patients and administering 2-MBq/kg body weight, for Method A, over time, one less patient dose would be available resulting in a need for another synthesis including all consequences (e.g., radiation exposure for the operator, costs for materials).

**Table 2:** Comparison of % RCY data obtained with Method C utilizing different lots of PSMA-11 material and a germanium-68/gallium-68 generator (1.85 GBq) at the end of shelf life (left data), at the start of shelf life (central data), and in the middle of shelf life (right data).

Method C									
**Generator calibration date**	2020/08/11			2021/06/22			2021/06/22		
**Lot PSMA-11 ID 9921.0001**	PSMA-06-19091803.01			PSMA-06-19091803.01			PSMA-06-20120102.07		
**Precursor reconstitution date**	2021/03/16			2021/06/29			2021/09/22		
**Process number**	**Date**	**RCY, %** **^a^**	**Radioactivity** **at EOS, MBq ^a^**	**Date**	**RCY, %** **^a^**	**Radioactivity** **at EOS, MBq ^a^**	**Date**	**RCY, %** **^a^**	**Radioactivity** **at EOS, MBq ^a^**
1	2021/03/16	**83**	442	2021/06/29	**80**	954	2021/09/22	**80**	772
2	2021/03/17	**84**	448	2021/06/30	**81**	961	2021/09/24	**79**	755
3	2021/03/24	**79**	416	2021/07/07	**81**	953	2021/09/29	**79**	748
4	2021/03/30	**81**	411	2021/07/14	**80**	924	2021/10/05	**82**	768
5	2021/03/31	**78**	401	2021/07/21	**81**	918	2021/10/06	**80**	740
6	2021/04/07	**79**	399	2021/07/27	**82**	909	2021/10/13	**79**	723
7	2021/04/21	**79**	387	2021/07/28	**82**	915	2021/10/20	**82**	740
8	2021/04/22	**78**	380	2021/08/03	**82**	899	2021/10/26	**81**	720
9	2021/05/05	**80**	372	2021/08/04	**80**	875	2021/10/27	**79**	700
10	2021/05/12	**78**	360	2021/08/31	**81**	826	2021/11/09	**80**	685
**Means**		**79.9**	**401.6**		**81.0**	**913.4**		**80.1**	**735.1**
**SD**		**2.13**	**28.62**		**0.82**	**40.77**		**1.20**	**28.23**
		**Between Groups**		**Within Groups**					
**SS-Sum of squares**		6.8667		59.8000					
**DF-degrees of freedom**		2		27					
**MS-Mean square**		3.4333		2.2148					
**Fisher F**		1.5502							
***p*-Value**		0.2305							

^a^ Non-decay corrected.

**Table 3:** Comparison of quality control data on [^68^Ga]Ga-PSMA-11 preparations obtained using different methods to prepare the precursor solution.

Method A					Method B				Method C			
**Generator calibration date:**	2020/08/11 ^a^				2020/08/11 ^b^				2020/08/11 ^c^			
**Process Number**	**Date**	**RCP, % ^d^**	**gallium-68 ion, %**	**Radioactivity** **on C18 Post-elution, MBq ^e^**	**Date**	**RCP, % ^d^**	**gallium-68 ion, %**	**Radioactivity on C18 Post-elution, MBq ^e^**	**Date**	**RCP, % ^d^**	**gallium-68 ion, %**	**Radioactivity on C18 Post-elution, MBq ^e^**
1	2020/11/10	96.92	0.53	32	2021/02/09	98.24	0.32	21	2021/03/16	98.20	0.15	8
2	2020/11/11	96.75	0.56	50	2021/02/10	95.86	0.73	47	2021/03/17	98.54	0.10	4
3	2020/11/12	96.49	0.71	37	2021/02/16	97.99	0.18	22	2021/03/24	97.93	0.28	21
4	2020/11/17	95.59	0.73	76	2021/02/17	97.76	0.30	25	2021/03/30	98.16	0.19	15
5	2020/11/18	97.01	0.33	50	2021/02/23	98.50	0.11	3	2021/03/31	97.78	0.38	19
6	2020/11/19	95.73	0.74	59	2021/03/02	98.60	0.27	11	2021/04/07	98.12	0.28	19
7	2020/11/24	95.32	0.93	61	2021/03/03	97.90	0.49	37	2021/04/21	97.79	0.22	16
8	2020/12/01	**95.09**	1.87	110	2021/03/09	98.61	0.05	7	2021/04/22	97.68	0.28	17
9	2020/12/02	**95.02**	1.32	94	2021/03/10	97.81	0.17	3	2021/05/05	97.63	0.22	23
10	2020/12/10	**95.01**	1.63	124		**n.d.**	**n.d.**		2021/05/12	98.02	0.18	11

^a^ Germanium-68/gallium-68 generator at the start of shelf life. ^b^ Germanium-68/gallium-68 generator in the middle of shelf life. ^c^ Germanium-68/gallium-68 generator at the end of shelf life. ^d^ According to the specifications given by the Ph. Eur. in the Monograph Gallium (^68^Ga) PSMA-11 injection (3044): the sum of [^68^Ga]Ga-PSMA-11 stereoisomer 1 and [^68^Ga]Ga-PSMA-11 stereoisomer 2 to be minimum 95% of the total radioactivity due to gallium-68. ^e^ Measured during the process with the detector included in the module.

**Table 4:** *T*-score values.

Method	Sample Size (n)	Mean	SD	Mean Difference (MD)	Pooled Variance	Pooled Standard Deviation (Sp)	*t*-Score	Degrees of Freedom
A	10	70.6	6.24	9.30	21.74	4.66	4.46	18
C	10	79.9	2.13					
A	10	70.6	6.24	8.96	27.92	5.28	3.69	17
B	9	79.6	3.94					
B	9	79.6	3.94	0.34	9.71	3.12	0.24	17
C	10	79.9	2.13					
**significance level of α**				0.05				
**t_α(0.05), df (18)_ value in the t-table**				2.10				
**t_α(0.05), df (17)_ value in the t-table**				2.11				

## Data Availability

The data presented in this study are available on request from the corresponding author.

## References

[1] European Directorate for the Quality of Medicines (2020). Monograph: 2902, Chemical precursor for radiopharmaceutical preparations. European Pharmacopeia.

[2] European Directorate for the Quality of Medicines (2020). Monograph: 2034, Substances for pharmaceutical use. European Pharmacopeia.

[3] Pijarowska-Kruszyna J., Garnuszek P., Decristoforo C., Mikołajczak R., Eppard E. (2021). Radiopharmaceutical Precursors for Theranostics. Book Theranostics—An Old Concept in New Clothing.

[4] Iudicello A., Genovese F., Di Iorio V., Cicoria G., Boschi S. (2021). An HPLC and UHPLC-HRMS approach to study PSMA-11 instability in aqueous solution. EJNMMI Radiopharm. Chem..

[5] Eppard E., Pèrez-Malo M., Rösch F. (2017). Improved radiolabeling of DOTATOC with trivalent radiometals for clinical application by addition of ethanol. EJNMMI Radiopharm. Chem..

[6] Luurtsema G., Pichler V., Bongarzone S., Seimbille Y., Elsinga P., Gee A., Vercouillie J. (2021). EANM guideline for harmonization on molar activity or specific activity of radiopharmaceuticals: Impact on safety and imaging quality. EJNMMI Radiopharm. Chem..

[7] Velikyan I. (2014). Prospective of 68Ga-radiopharmaceutical development. Theranostics.

[8] (2021). Monograph no. 3044 “Gallium (^68^Ga) PSMA-11 Injection”.

[9] Eder M., Neels O., Müller M., Bauder-Wüst U., Remde Y., Schäfer M., Hennrich U., Eisenhut M., Afshar-Oromieh A., Haberkorn U. (2014). Novel preclinical and radiopharmaceutical aspects of Ga-PSMA-HBED-CC: A new PET tracer for imaging of prostate Cancer. Pharmaceuticals.

[10] Eder M., Schäfer M., Bauder-Wüst U., Hull W.E., Wängler C., Mier W., Haberkorn U., Eisenhut M. (2012). 68Ga-complex lipophilicity and the targeting property of a urea-based PSMA inhibitor for PET imaging. Bioconjug. Chem..

[11] Eder M., Wängler B., Knackmuss S., LeGall F., Little M., Haberkorn U., Mier W., Eisenhut M. (2008). Tetrafluorophenolate of HBED-CC: A versatile conjugation agent for 68Ga-labeled small recombinant antibodies. Eur. J. Nucl. Med. Mol. Imaging.

[12] Meisenheimer M., Kürpig S., Essler S., Eppard E. (2020). Manual vs automa ted 68Ga-radiolabelling. A comparison of optimized processes. J. Label Compd. Radiopharm..

[13] Vichi S., Infantino A., Cicoria G., Pancaldi D., Mostacci D., Lodi F., Marengo M. (2016). An innovative gamma-ray spectrometry system using a compact and portable CZT detector for radionuclidic purity tests of PET radiopharmaceuticals. Radiat. Eff. Defects Solids.

[14] International Conference of Harmonization of Technical Requirements for Pharmaceuticals for Human Use. Guidelines for Stability 2011. https://www.ich.org/products/guidelines.

[15] Imberti C., Chen Y.-L., Foley C.A., Ma M.T., Paterson B.M., Wang Y., Young J.D., Hiderb R.C., Blower P.J. (2019). Tuning the properties of tris (hydroxypyridinone) ligands: Efficient 68Ga chelators for PET imaging. Dalton Trans..

[16] Wadas T.J., Wong E.H., Weisman G.R., Anderson C.J. (2010). Coordinating Radiometals of copper, gallium, indium, yttrium, and zirconium for PET and SPECT imaging of disease. Chem. Rev..

